# Supplementary material for: Self-Assembled Matrine-PROTAC Encapsulating Zinc(II) Phthalocyanine with GSH-Depletion-Enhanced ROS Generation for Cancer Therapy
Source: Molecules. 2024 Apr 18;29(8):1845. doi: 10.3390/molecules29081845 (PMC11054886; doi:10.3390/molecules29081845)
Supplement: Supplementary file 1 [file molecules-29-01845-s001.zip › molecules-2870113-supplementary.pdf]

Self-assembled matrine-PROTAC encapsulating zinc(II) phthalocyanine with GSH depletion enhanced ROS generation for cancer therapy

Sitong Lai [1,†], Bing Wang [2,†], Kunhui Sun [2], Fan Li [1], Qian Liu [1], Xie-an Yu [2], Lihe Jiang [3,4*] and Lisheng Wang [1,*]

[1] School of medicine, Guangxi University, Nanning, 530004, China; LaiSitong0718@163.com (S.L.); lifanffff@163.com; (F.L.); lq622727@126.com (Q.L.)
[2] NMPA Key Laboratory for Quality Research and Evaluation of Traditional Chinese Medicine, Shenzhen Institute for Drug Control, Shenzhen 518057, China; wangbingszyj@163.com (B.W.); sunkunhuilst@163.com (K.S.)
[3] School of Basic Medical Sciences, Youjiang Medical University for Nationalities, Baise, 533000, Guangxi, P.R. China
[4] Shenzhen Key Laboratory of Southern Subtropical Plant Diversity, Fairylake Botanical Garden, Shenzhen & Chinese Academy of Science, Shenzhen 518004, China
* Correspondence: jianglihe@ymun.edu.cn (L.J.); lswang@gxu.edu.cn (L.W.)
† These authors contributed equally to this work.

1. Supplementary Figures

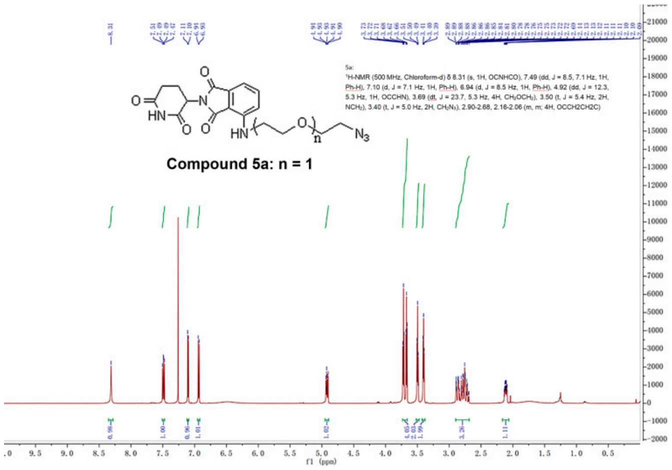

Figure S1. ^{1}H-NMR spectrum of compound 5a (The solvent is Chloroform-d).

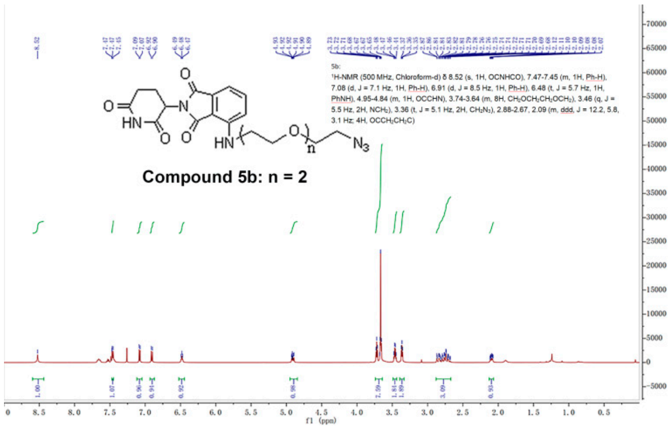

Figure S2. ^{1}H-NMR spectrum of compound 5b (The solvent is Chloroform-d).

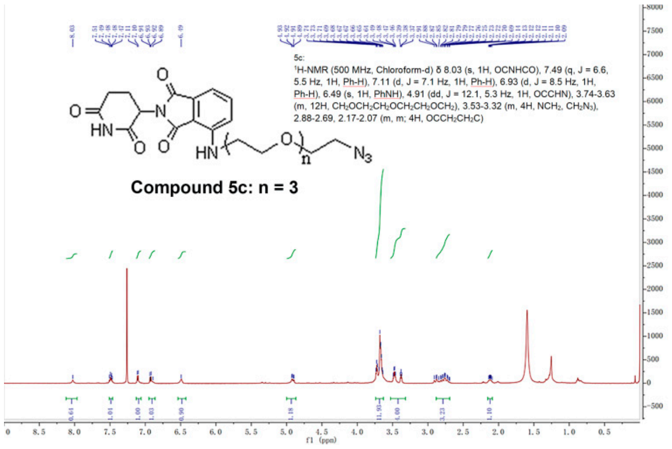

Figure S3. ^{1}H-NMR spectrum of compound 5c (The solvent is Chloroform-d).

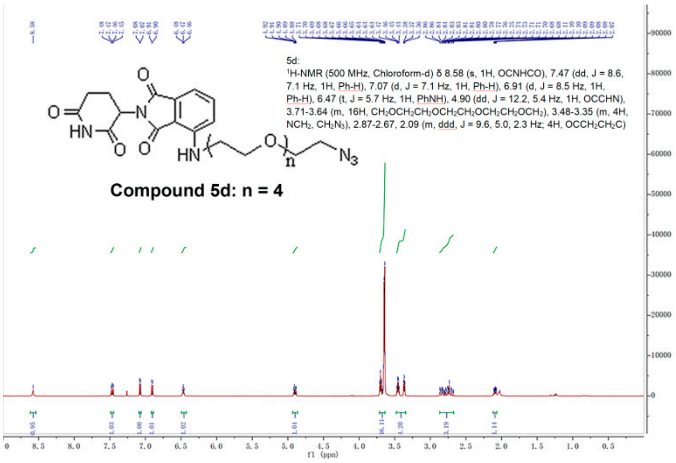

Figure S4. ^{1}H-NMR spectrum of compound 5d (The solvent is Chloroform-d).

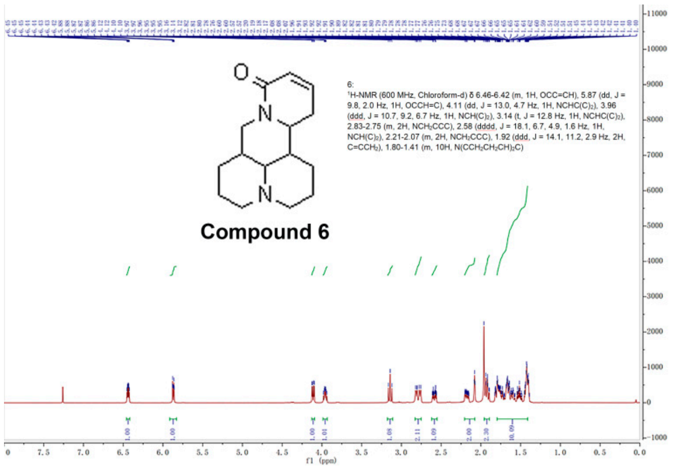

Figure S5. ^{1}H-NMR spectrum of compound 6 (The solvent is Chloroform-d).

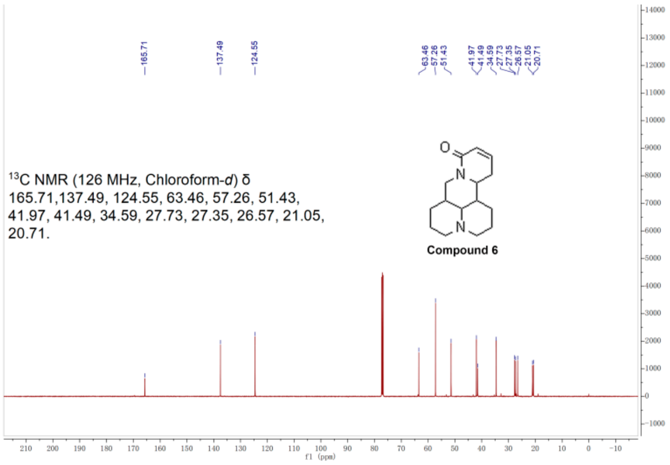

Figure S6. ^{13}C-NMR spectrum of compound 6 (The solvent is Chloroform-d).

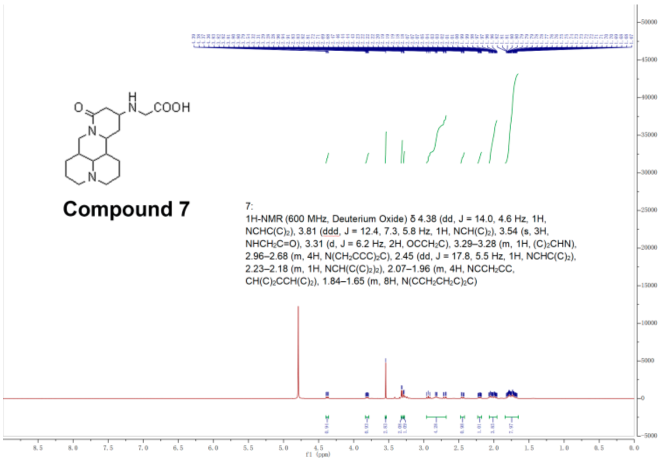

Figure S7. ^{1}H-NMR spectrum of compound 7 (The solvent is Deuterium Oxide).

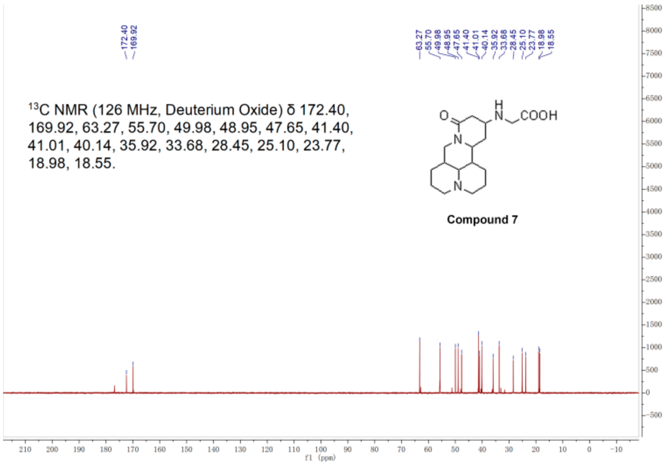

Figure S8. ^{13}C-NMR spectrum of compound 7 (The solvent is Deuterium Oxide).

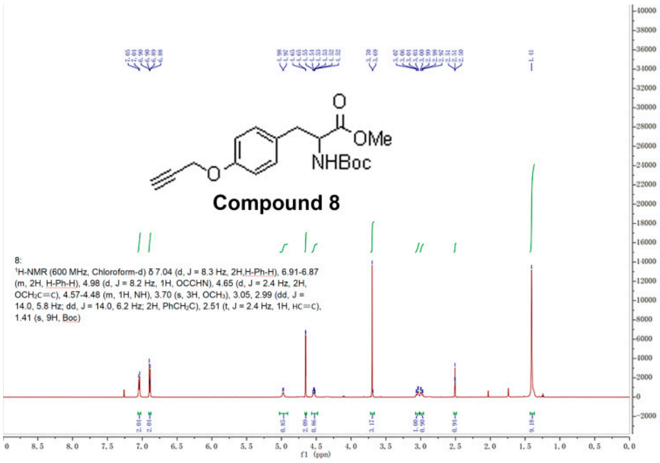

Figure S9. ^{1}H-NMR spectrum of compound 8 (The solvent is Chloroform-d).

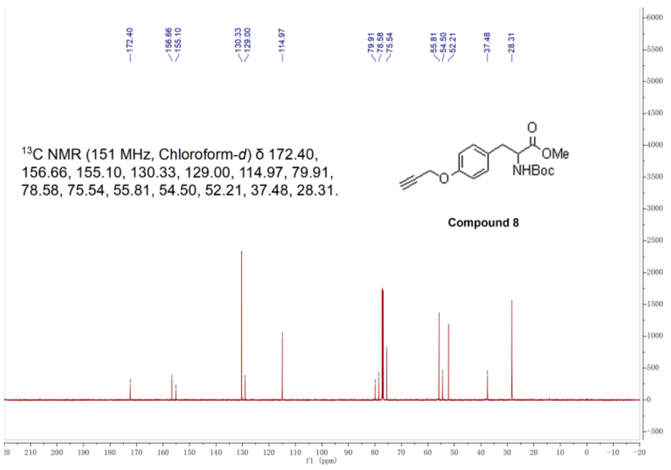

Figure S10. ^{13}C-NMR spectrum of compound 8 (The solvent is Chloroform-d).

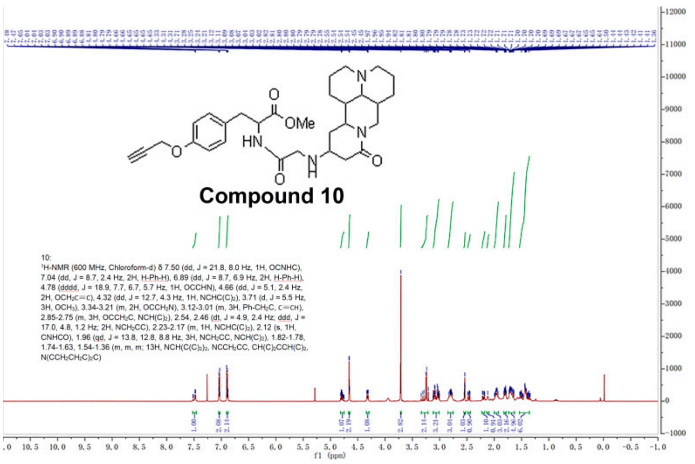

Figure S11. ^{1}H-NMR spectrum of compound 10 (The solvent is Chloroform-d).

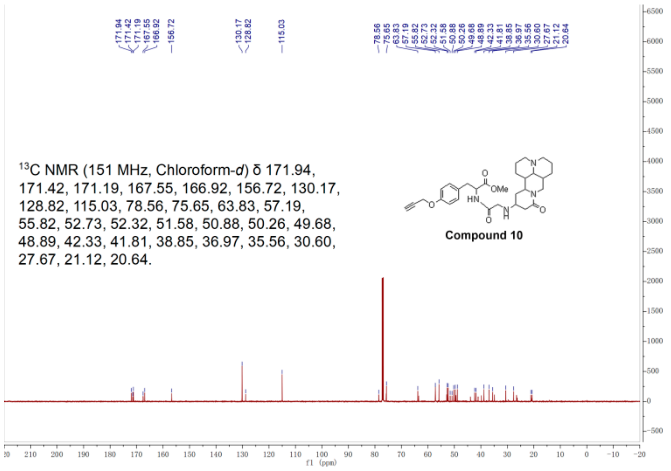

Figure S12. ^{13}C-NMR spectrum of compound 10 (The solvent is Chloroform-d).

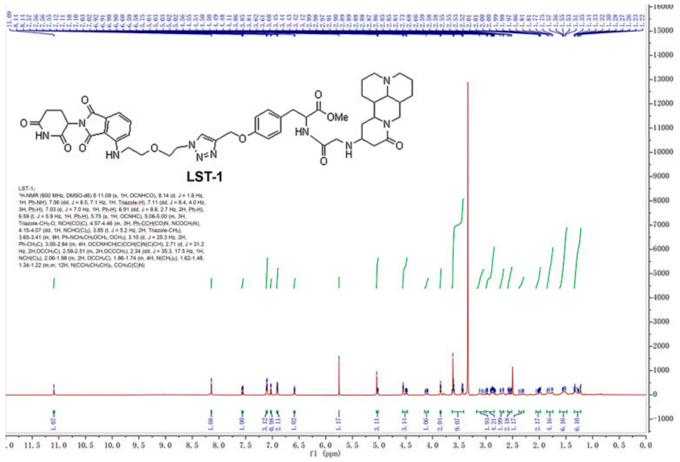

Figure S13. ^{1}H-NMR spectrum of LST-1 (The solvent is DMSO-d6).

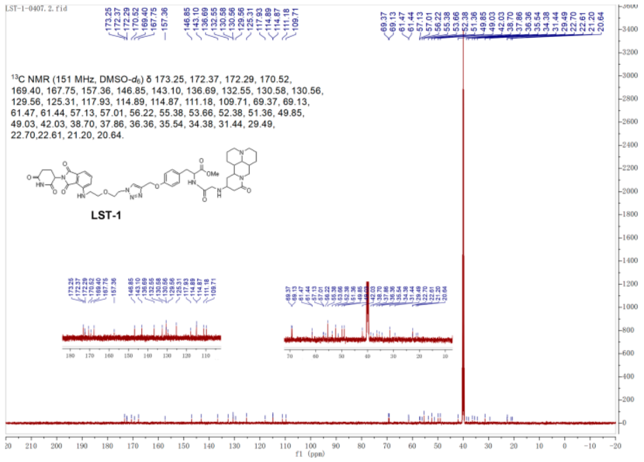

Figure S14. ^{13}C-NMR spectrum of LST-1 (The solvent is DMSO-d6).

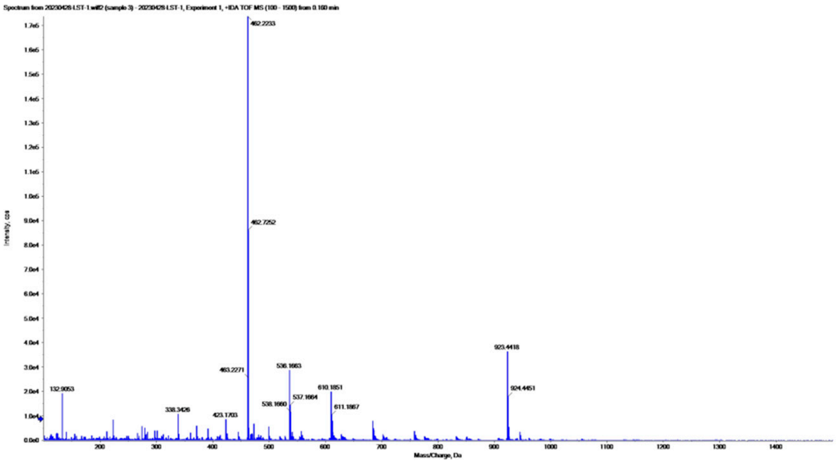

Figure S15. HRMS of LST-1.

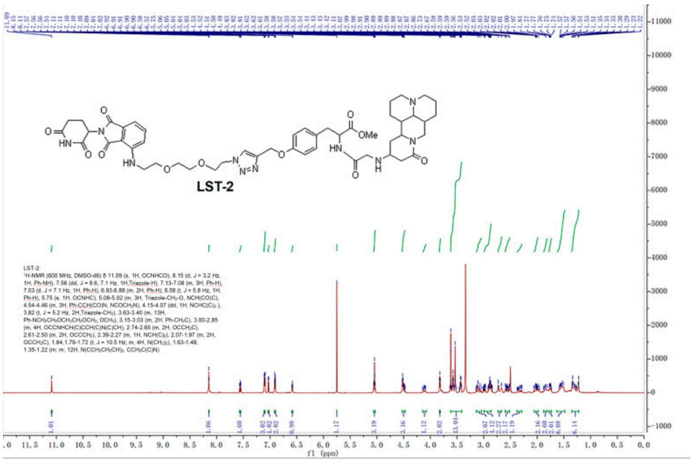

Figure S16. ^{1}H-NMR spectrum of LST-2 (The solvent is DMSO-d6).

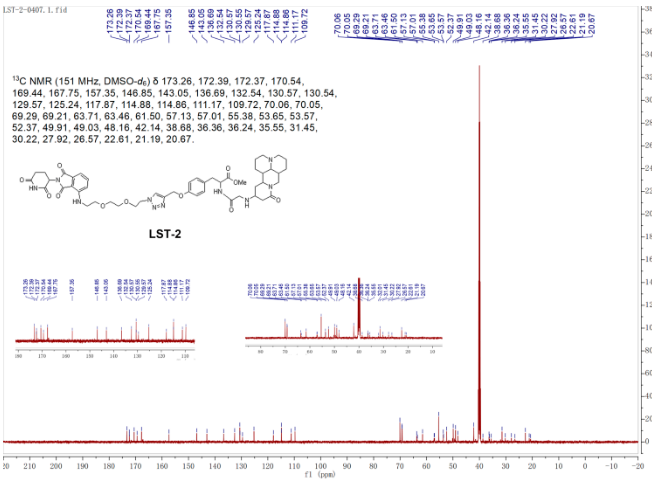

Figure S17. ^{13}C-NMR spectrum of LST-2 (The solvent is DMSO-d6).

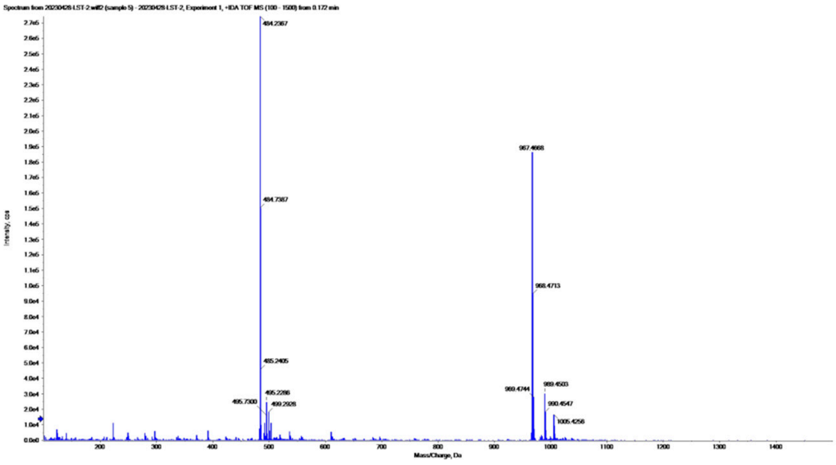

Figure S18. HRMS of LST-2.

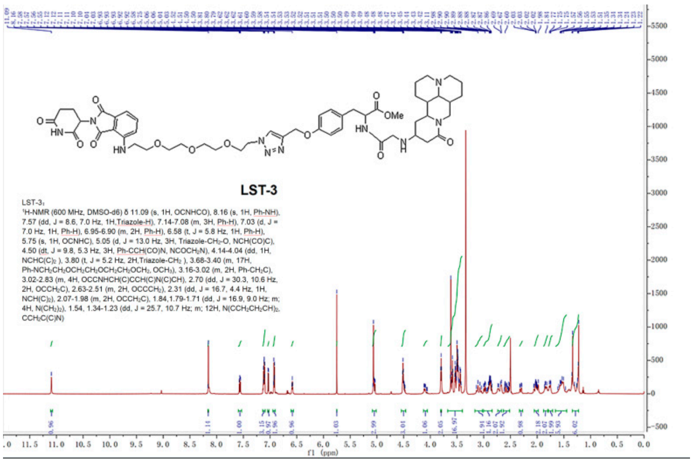

Figure S19. ^{1}H-NMR spectrum of LST-3 (The solvent is DMSO-d6).

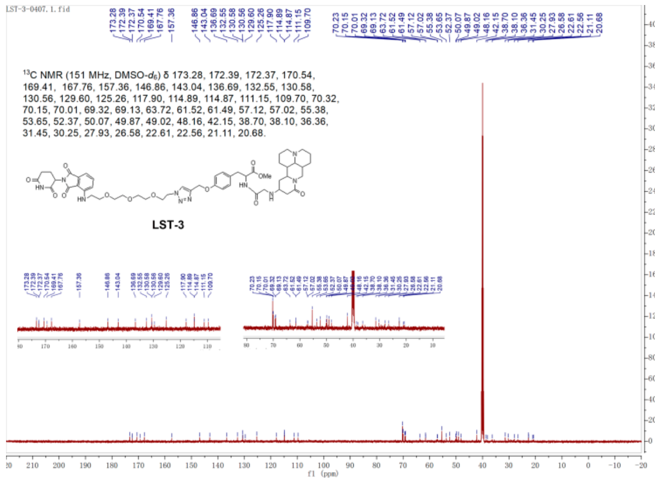

Figure S20. ^{13}C-NMR spectrum of LST-3 (The solvent is DMSO-d6).

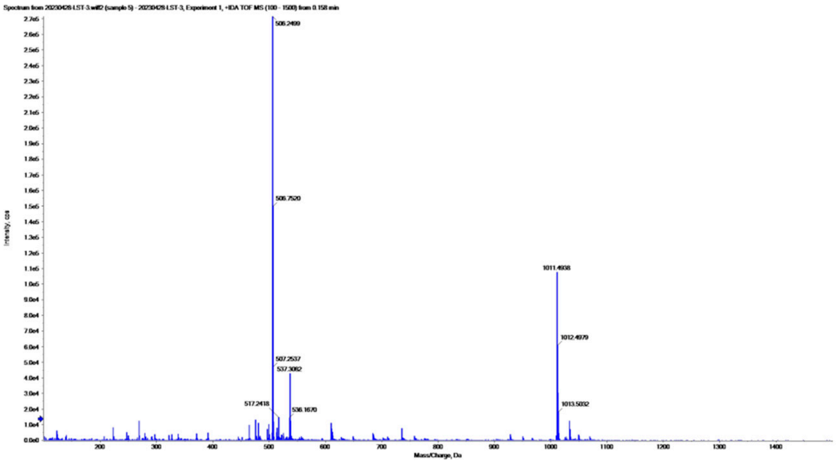

Figure S21. HRMS of LST-3.

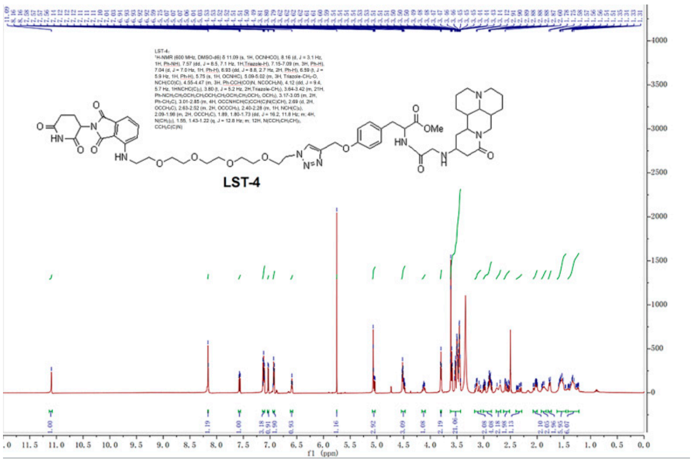

Figure S22. ^{1}H-NMR spectrum of LST-4 (The solvent is DMSO-d6).

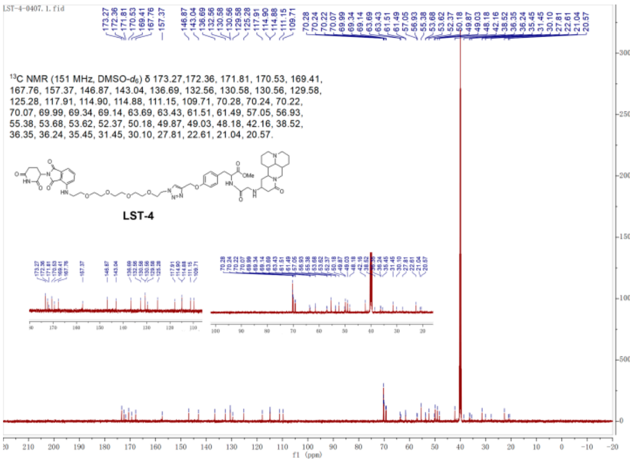

Figure S23. ^{13}C-NMR spectrum of LST-4 (The solvent is DMSO-d6).

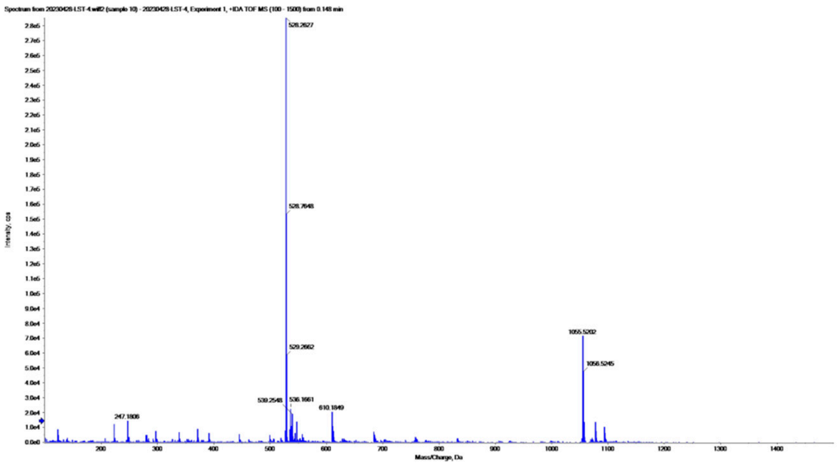

Figure 24. HRMS of LST-4.

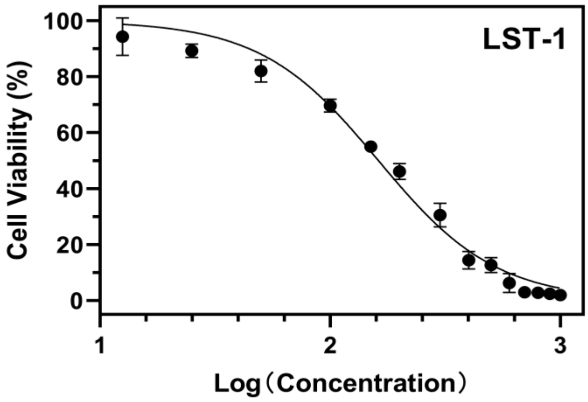

Figure S25. The dose-dependent inhibition curves of LST-1 (The unit of concentration: μM) (n=4).

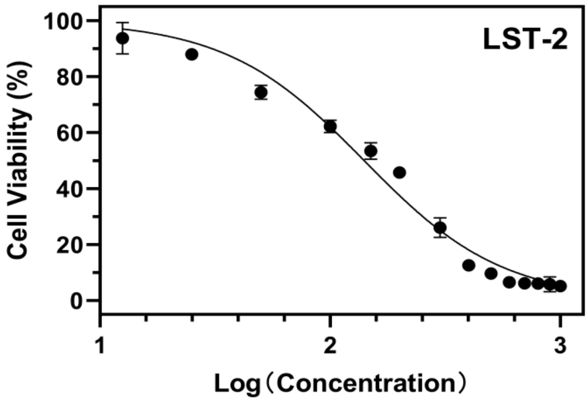

Figure S26. The dose-dependent inhibition curves of LST-2 (The unit of concentration: μM) (n=4).

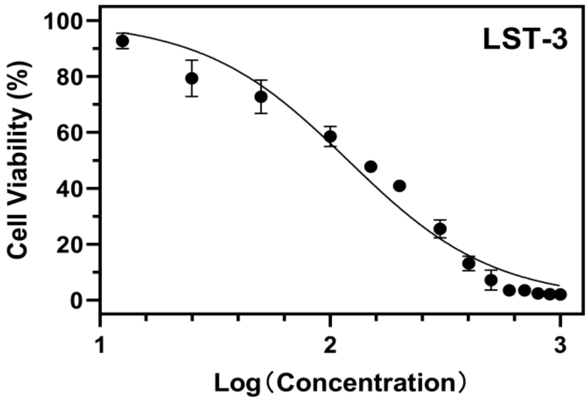

Figure S27. The dose-dependent inhibition curves of LST-3 (The unit of concentration: μM) (n=4).

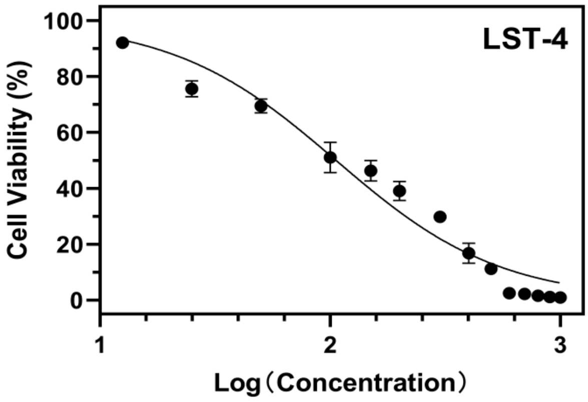

Figure S28. The dose-dependent inhibition curves of LST-4 (The unit of concentration: μM) (n=4).

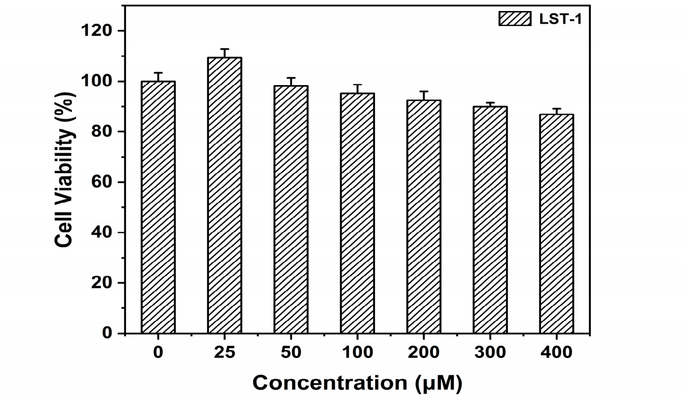

Figure S29. Viability of L02 cells treated with various concentrations of LST-1 (n=4).

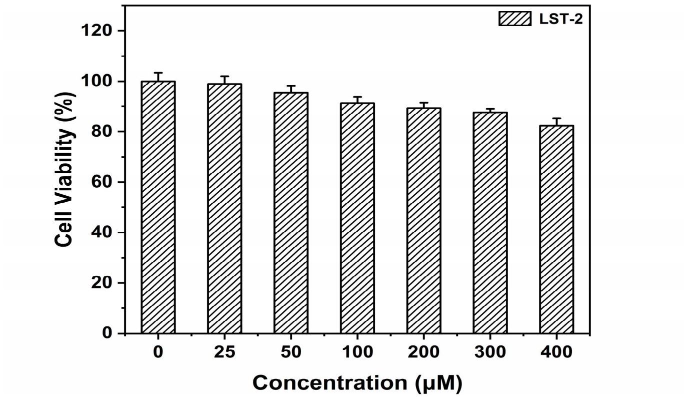

Figure S30. Viability of L02 cells treated with various concentrations of LST-2 (n=4).

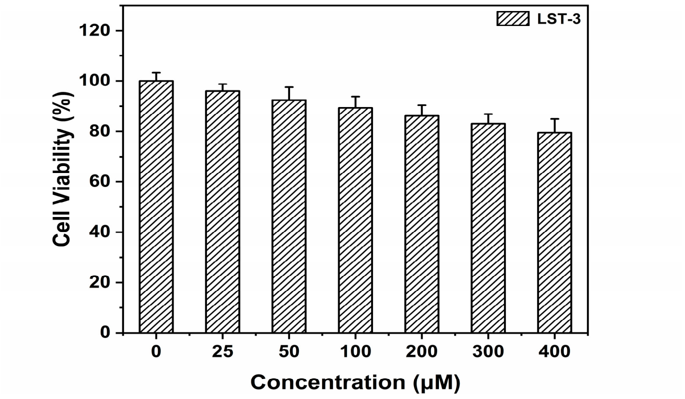

Figure S31. Viability of L02 cells treated with various concentrations of LST-3 (n=4).

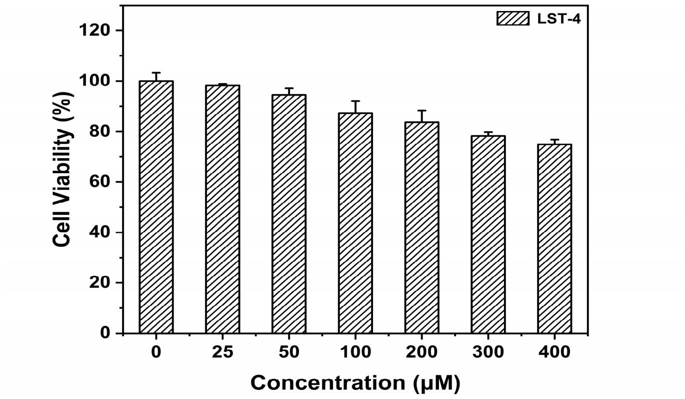

Figure S32. Viability of L02 cells treated with various concentrations of LST-4 (n=4).

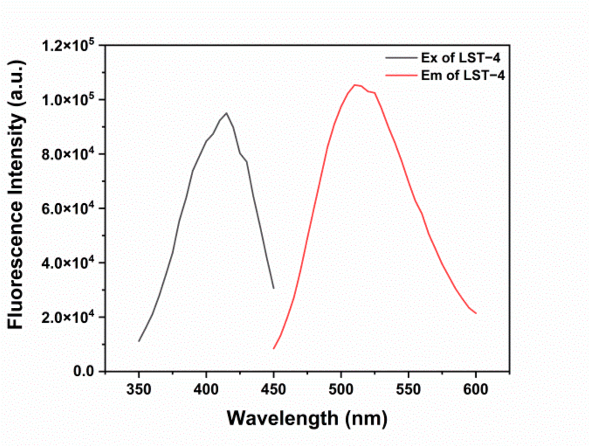

Figure S33. Excitation and emission wavelengths of LST-4 from a microplate reader.

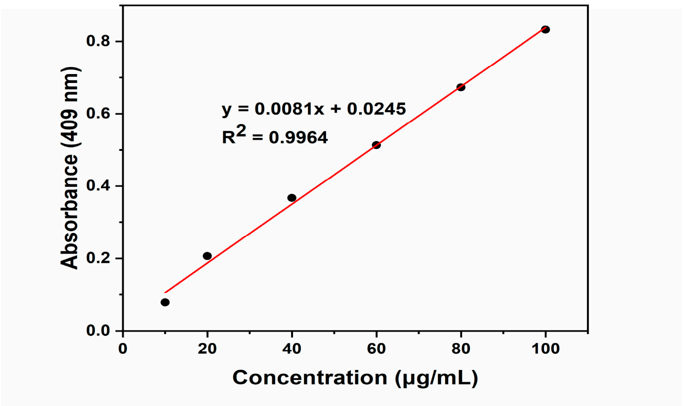

Figure S34. The liner plot of the absorbance of LST-4 in the wavelength of the maximum absorption as a function of concentration.

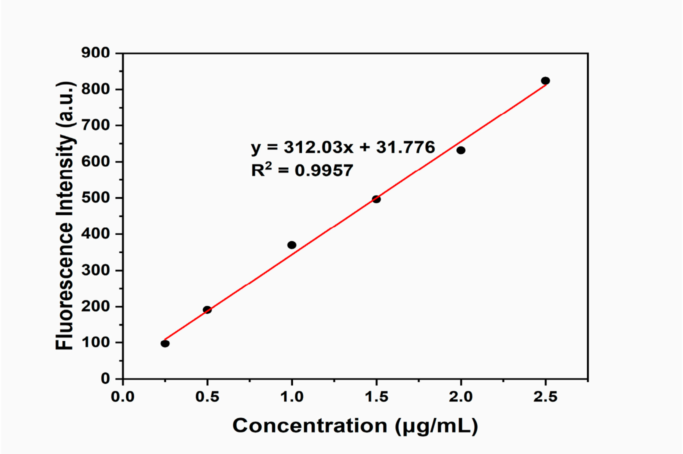

Figure S35. The line graph of the FL intensity of LST-4 with λ_{Ex} = 503 nm as a function of concentration.

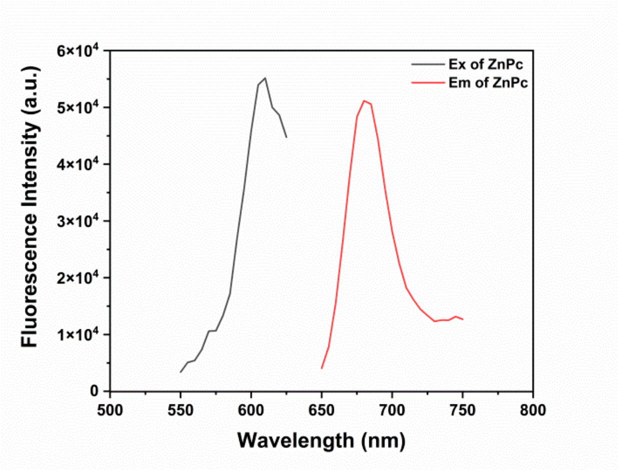

Figure S36. Excitation and emission wavelengths of ZnPc from a microplate reader.

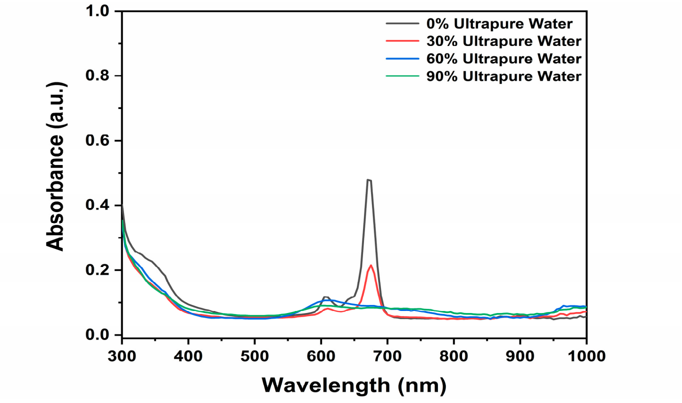

Figure S37. UV-Vis absorption spectra of ZnPc in water/DMSO mixtures with different water fractions.

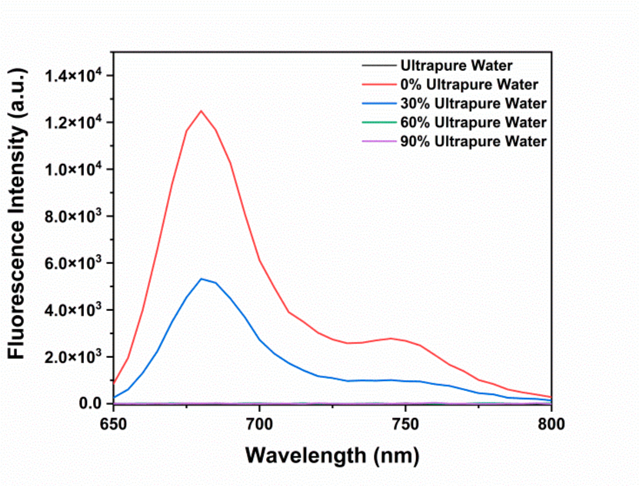

Figure S38. Fluorescence spectra of ZnPc in water/DMSO mixtures with different water fractions.

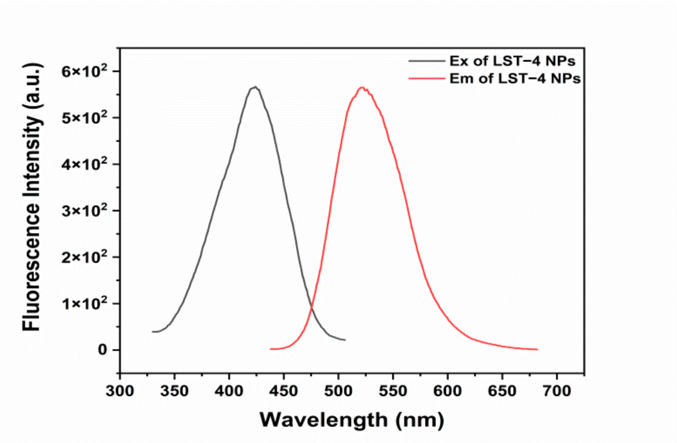

Figure S39. Excitation and emission wavelengths of LST-4 NPs from UV-2450 spectrophotometer (Shimadzu, Tokyo, Japan).

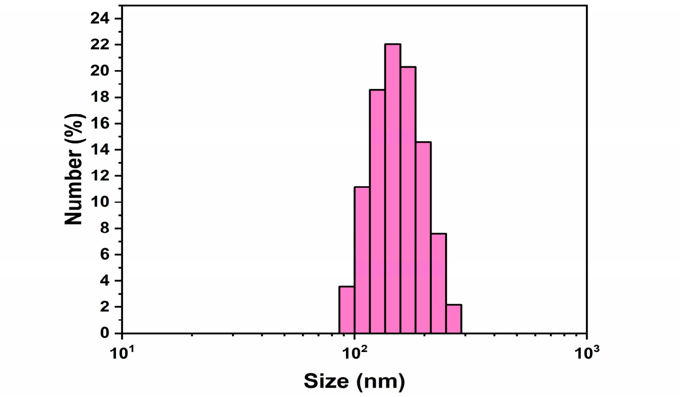

Figure S40. Size distribution of LST-4 NPs.

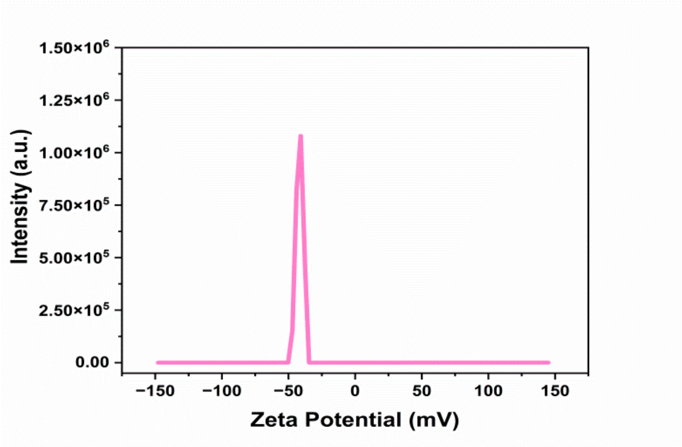

Figure S41. Zeta potentials of LST-4 NPs.

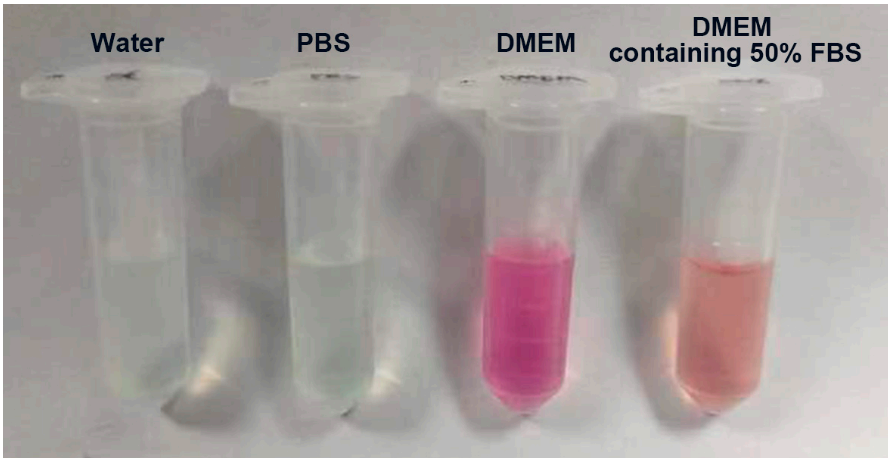

Figure S42. Solution state of LST-4 NPs incubated in water, PBS, DMEM and 50% FBS for 24 h.

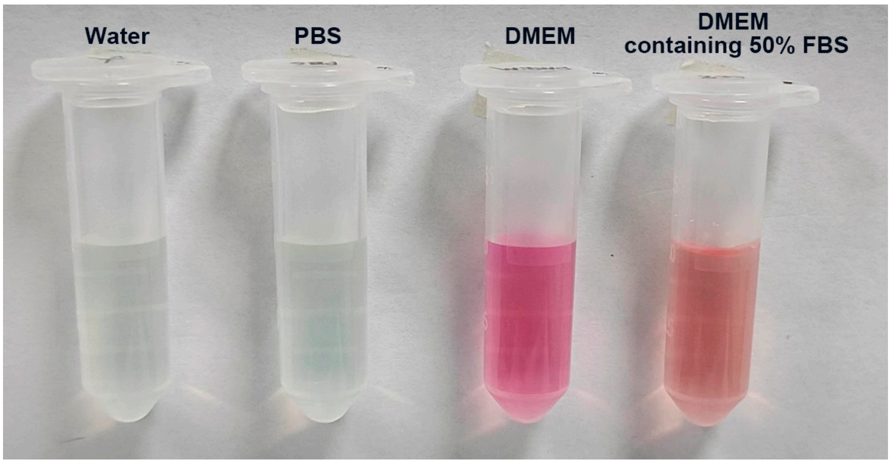

Figure S43. Solution state of LST-4@ZnPc NPs incubated in water, PBS, DMEM and 50% FBS for 24 h.

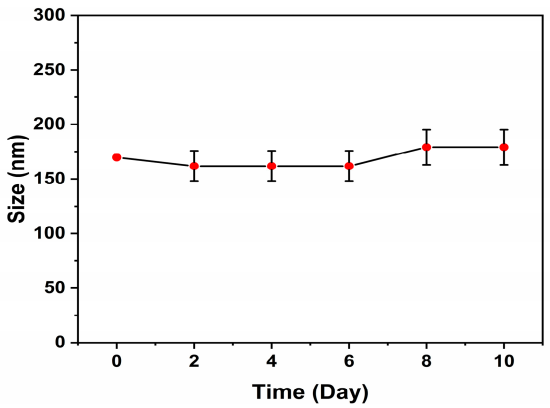

Figure S44. The measurement of particle size of LST-4@ZnPc NPs within 10 days under 4°C (n=3).

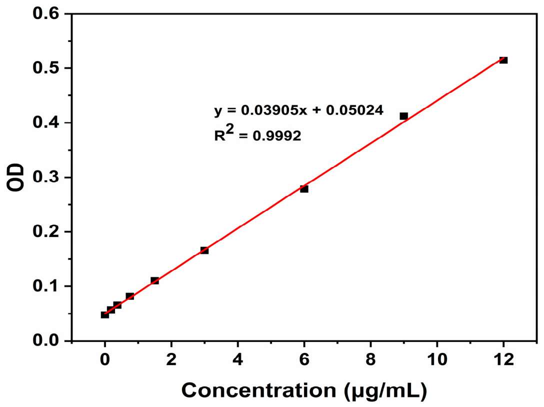

Figure S45. Standard curve of ZnPc in 5:1 (v/v) DMSO:water mixture (n=3).

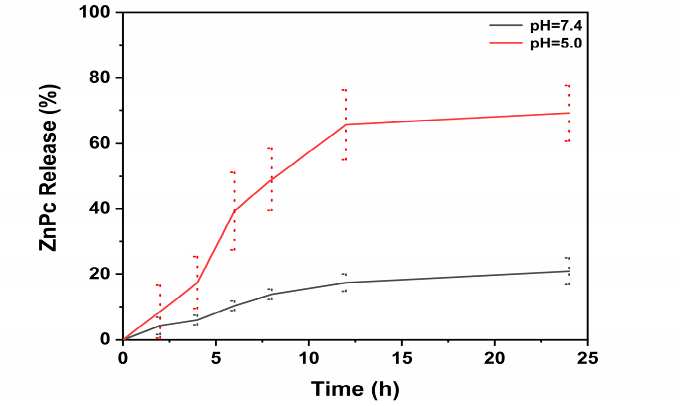

Figure S46. Release of ZnPc from LST-4@ZnPc NPs at pH 7.4 and 5.0 (n=3).

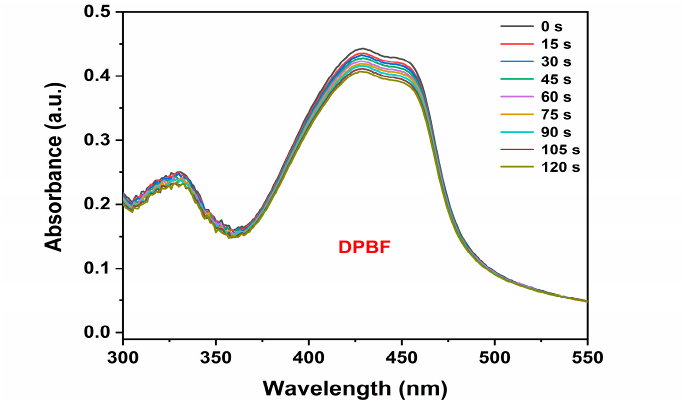

Figure S47. The UV–vis spectra of DPBF in DMSO for different irradiation times under 690 nm laser irradiation (0.2 W/cm^2).

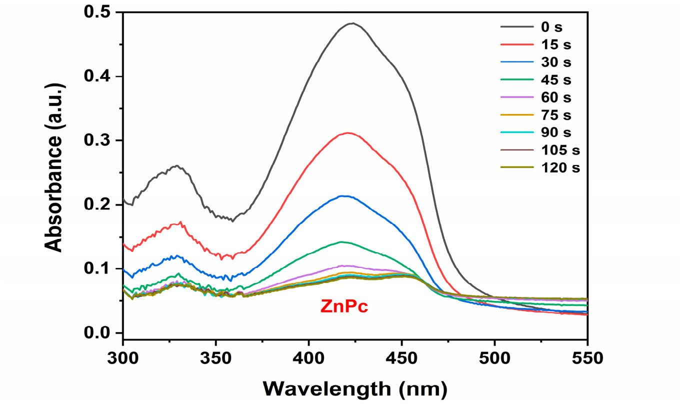

Figure S48. The UV-Vis spectra of DPBF containing ZnPc for different irradiation times.

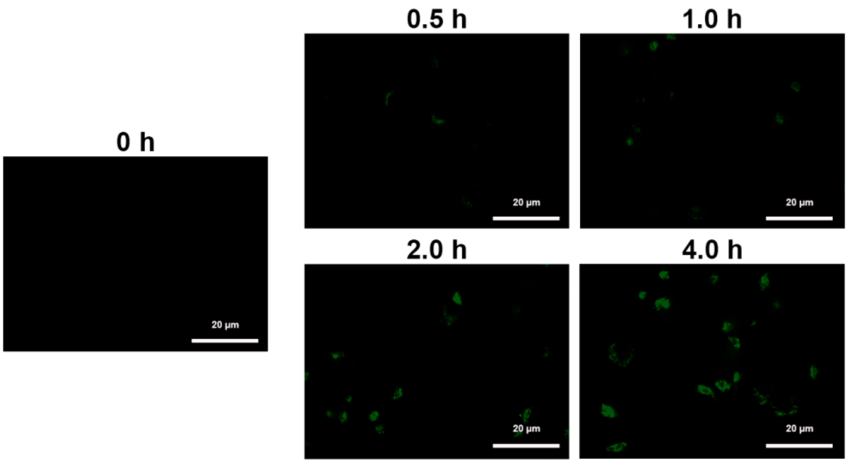

Figure S49. Cell uptake of LST-4 NPs by HepG2 cells with for different incubation times (0, 0.5, 1, 2, 4 h). The green fluorescence represents LST-4 NPs. (Scale bar represents 20 μm.)

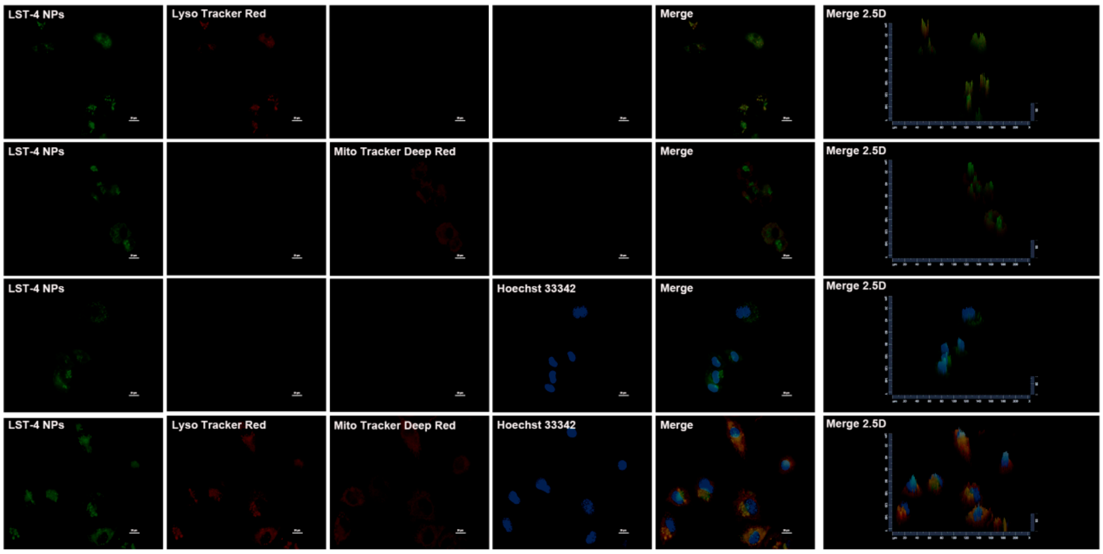

Figure S50. Confocal fluorescence images of HepG2 cells stained with LysoTracker Red, Mito Tracker Deep Red and Hoechst 33342 following incubation with LST-4 NPs for 4 h. (Scale bar represents 20 μm.)

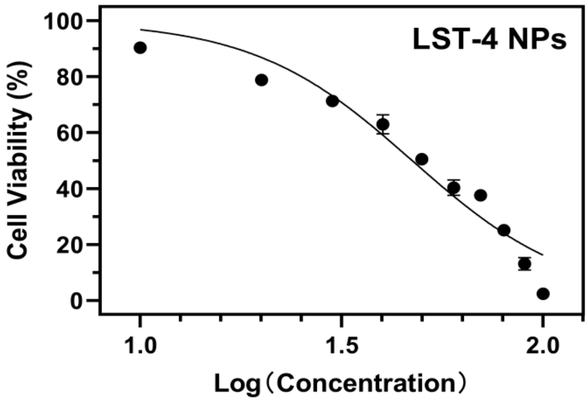

Figure S51. The dose-dependent inhibition curves of LST-4 NPs (The unit of concentration: μg/mL) (n=4).

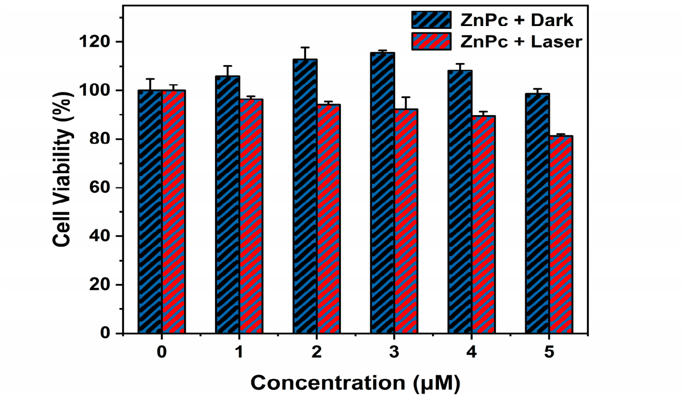

Figure S52. Viability of HepG2 cells treated with various concentrations of ZnPc in dark or upon exposure to laser radiation (a 690 nm laser (0.2 W/cm^2, 5 min)) (n=4).

.

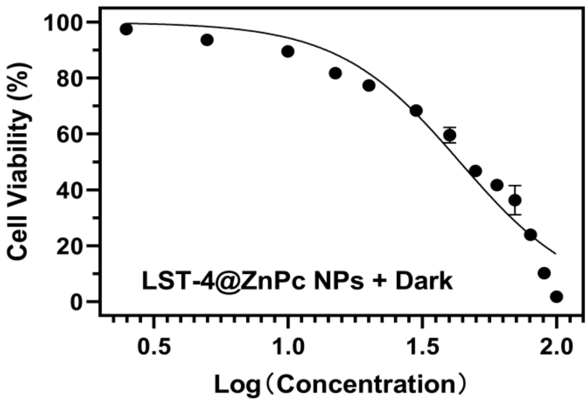

Figure S53. The dose-dependent inhibition curves of LST-4@ZnPc NPs in dark (The unit of concentration: μg/mL) (n=4).

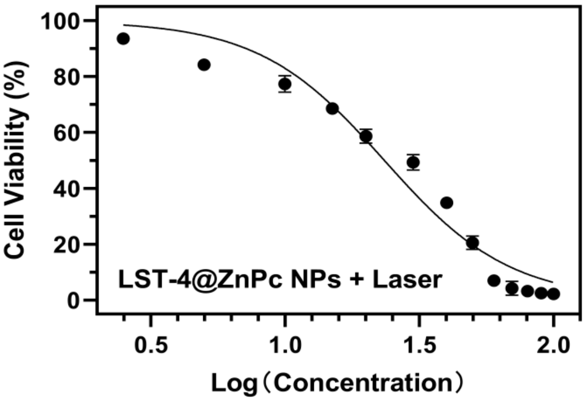

Figure S54. The dose-dependent inhibition curves of LST-4@ZnPc NPs upon exposure to laser radiation (a 690 nm laser (0.2 W/cm^2, 5 min)) (The unit of concentration: μg/mL) (n=4).

Table S1. Loading Efficiency of ZnPc in LST-4@ZnPc NPs.

No.	C_{ZnPc} (μg/mL)	LE (%)	Average (%)	RSD (%)
1	1.0464	71.75		
2	1.0976	75.262	74.442	1.95
3	1.1129	76.315		
